# Supplementary figures and images for: Dietary restriction improves intestinal cellular fitness to enhance gut barrier function and lifespan in D. melanogaster
Source: PLoS Genet. 2018 Nov 1;14(11):e1007777. doi: 10.1371/journal.pgen.1007777 (PMC6233930; doi:10.1371/journal.pgen.1007777)

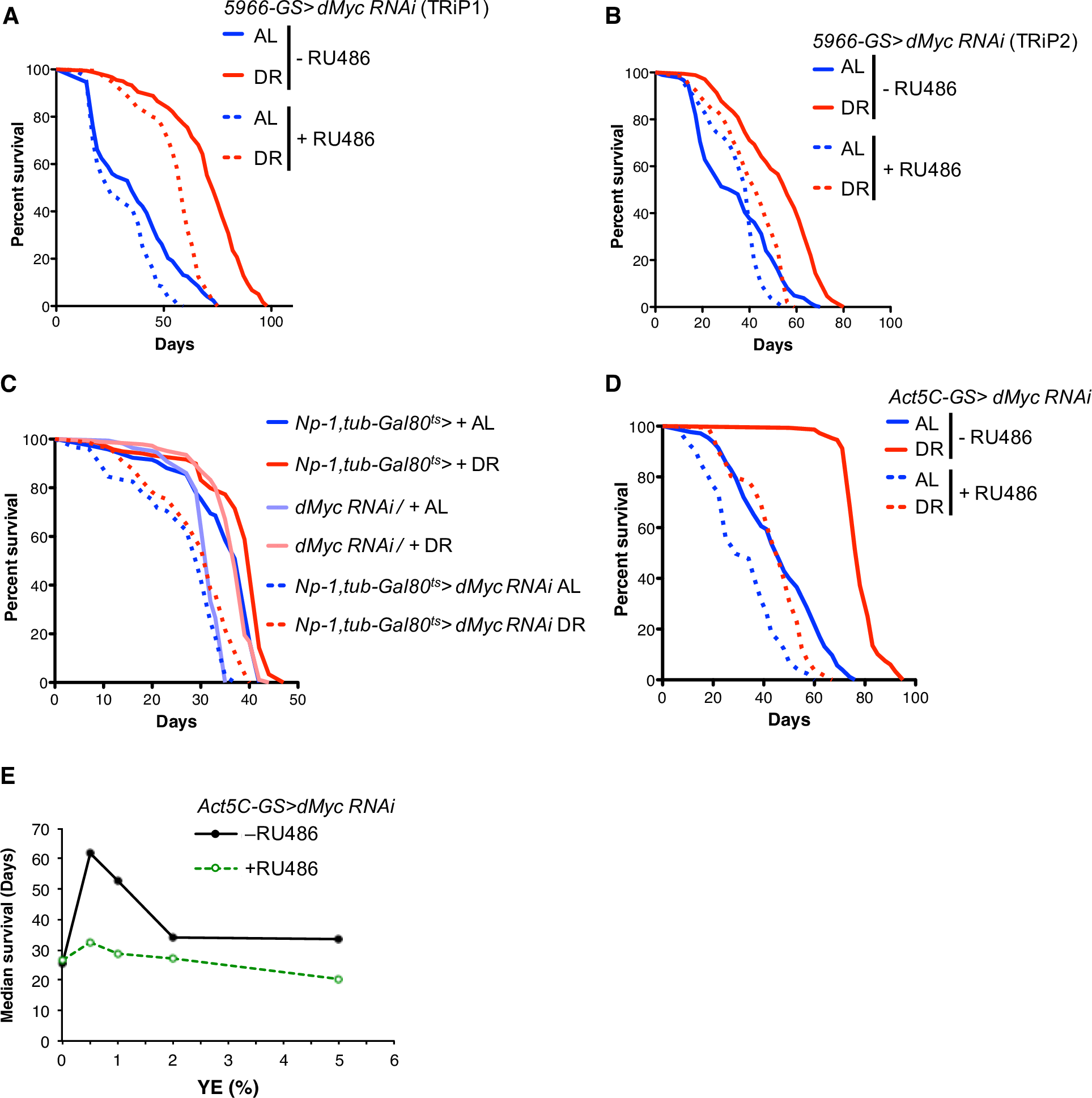

Supplement: S1 Fig — (A and B) Kaplan Meier survival analysis of 5966-GS>dMyc RNAi flies upon AL and DR using the other two different RNAi strains. (C) Kaplan Meier survival analysis of EC-specific dMyc knockdown (using Np-1-Gal4, tub-Gal80ts) upon AL and DR at 29°C. (D) Kaplan Meier survival analysis of ubiquitous knockdown of dMyc (using Act5C-GS-Gal4) upon AL and DR. (E) Median lifespan was calculated from Kaplan Meier survival analysis of ubiquitous knockdown of dMyc (using Act5C-GS-Gal4) under 5 different yeast extract conditions. Statistical analysis of the survival curves and the number of flies are provided in S2 Table. (TIF) [file pgen.1007777.s001.tif]

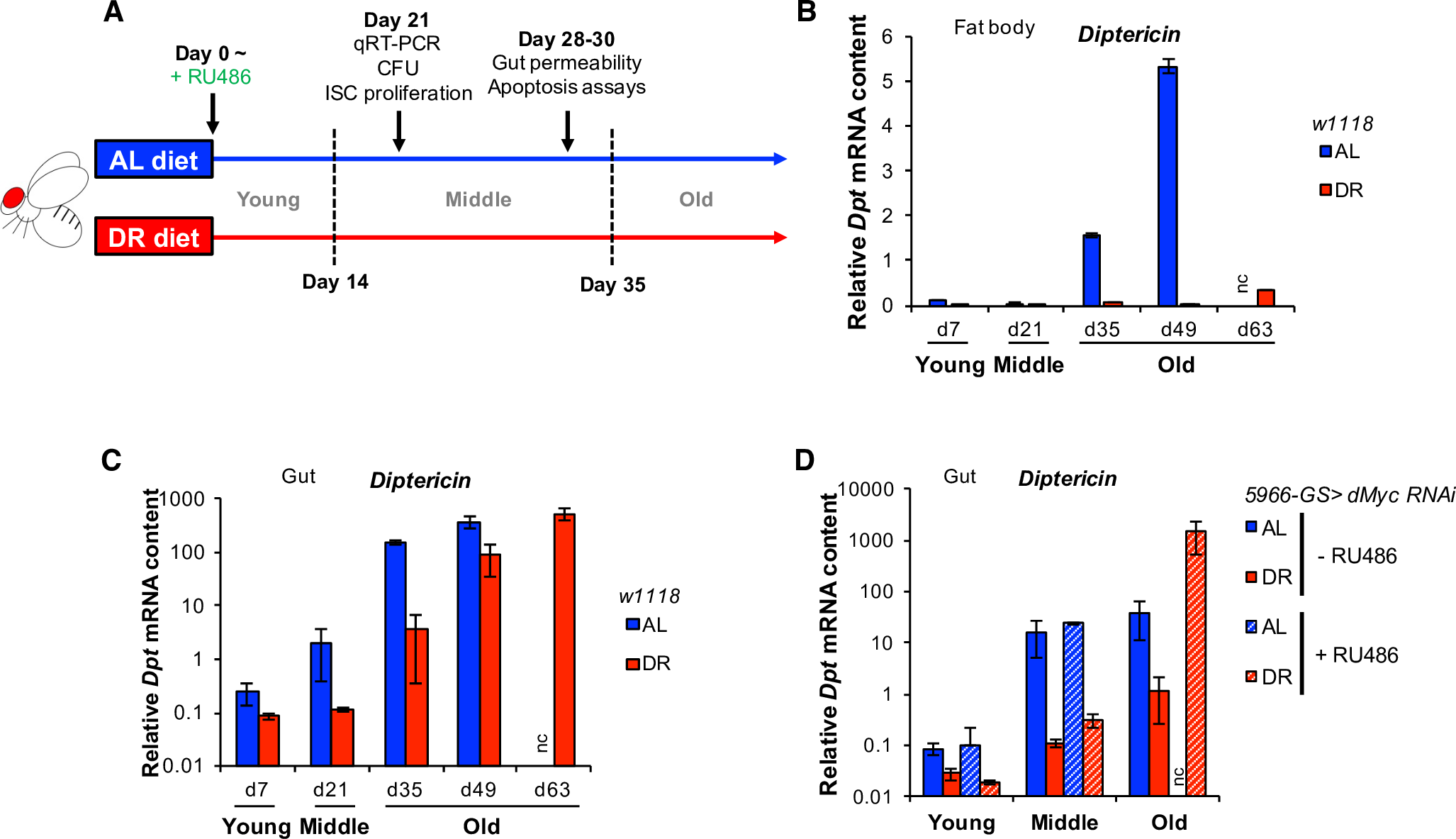

Supplement: S2 Fig — (A) Experimental timeline for 5966-GS>dMyc RNAi flies. (B and C) Diptericin (Dpt) mRNA expression in dissected fat bodies (B) and guts (C) from w1118 upon AL and DR was measured with age. mRNA expression from flies at day 0 was set to 1. (D) Age-dependent changes in mRNA expression of Diptericin in dissected guts in 5966-GS>dMyc RNAi flies. Young, middle and old represent day 7, 21 and 35 of ages, respectively. mRNA expression for flies at day 0 was set to 1. ‘nc’ represents samples that were not collected. (B-D) Error bars indicate SD from 3 independent biological replicates. (TIF) [file pgen.1007777.s002.tif]

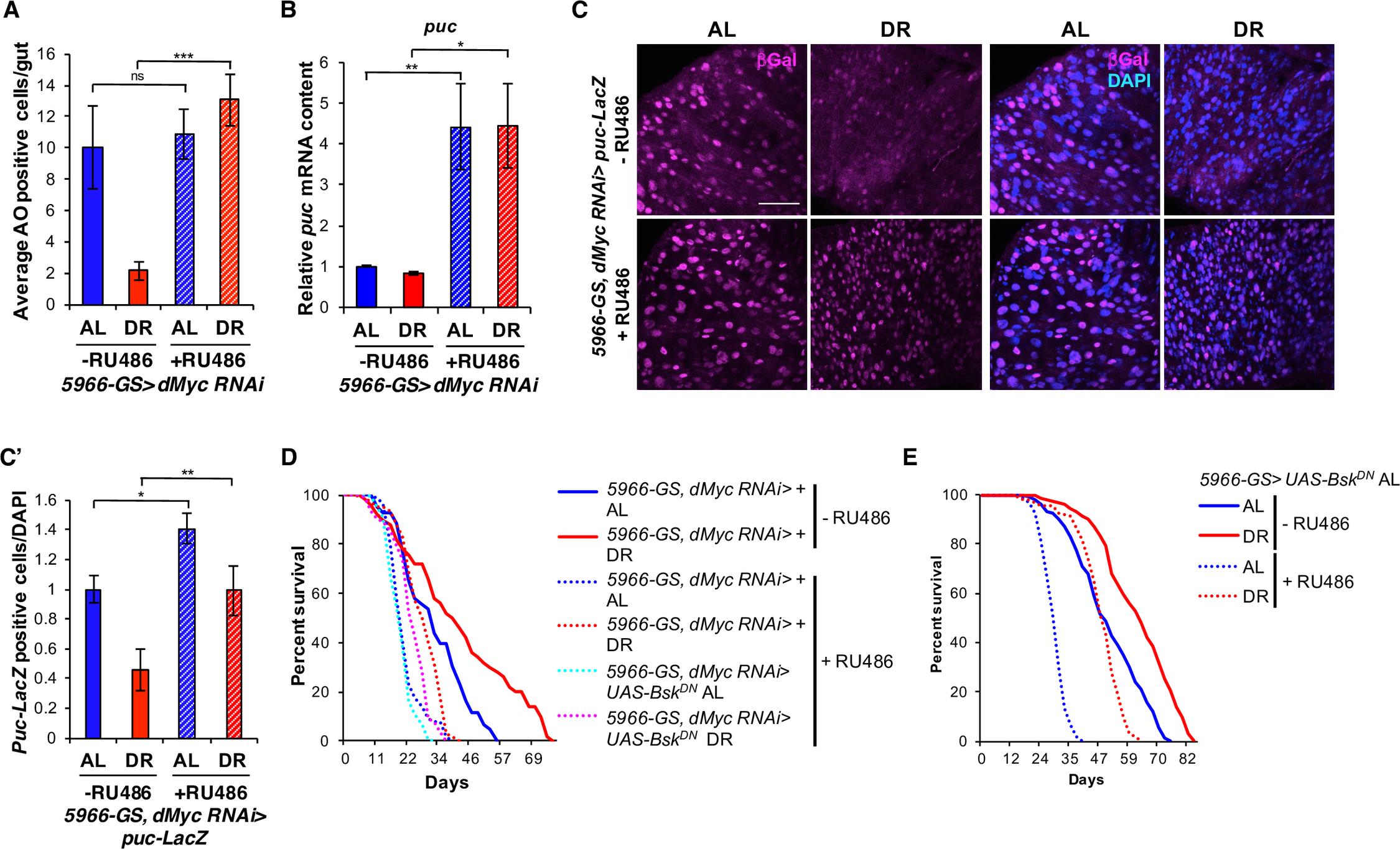

Supplement: S3 Fig — (A) Acridine orange staining using dissected guts from 30 day old 5966-GS>dMyc RNAi flies. Error bars indicate SEM of 12 guts. (*** p < 0.001 by t-test). (B) puc mRNA expression in dissected guts of 21 day old 5966-GS>dMyc RNAi flies. (C) Lac Z staining of dissected guts from 21 day old 5966-GS, dMyc RNAi; pucE69 (puc-lac Z) flies. Representative image (n = 11). Scale bar indicates 50 μm. (C’) Quantification of puc-lacZ positive cells from 11 images. (** p < 0.01, * p < 0.05 by t-test). (D) Kaplan-Meier survival analysis of 5966-GS, dMyc RNAi; + flies and 5966-GS, dMyc RNAi; UAS-BskDN flies upon AL and DR. (E) Kaplan Meier survival analysis of EBs/ECs-specific JNK inhibition (5966-GS> UAS-BskDN) upon AL and DR. Statistical analysis of the survival curves and number of flies are provided in S2 Table. (TIF) [file pgen.1007777.s003.tif]

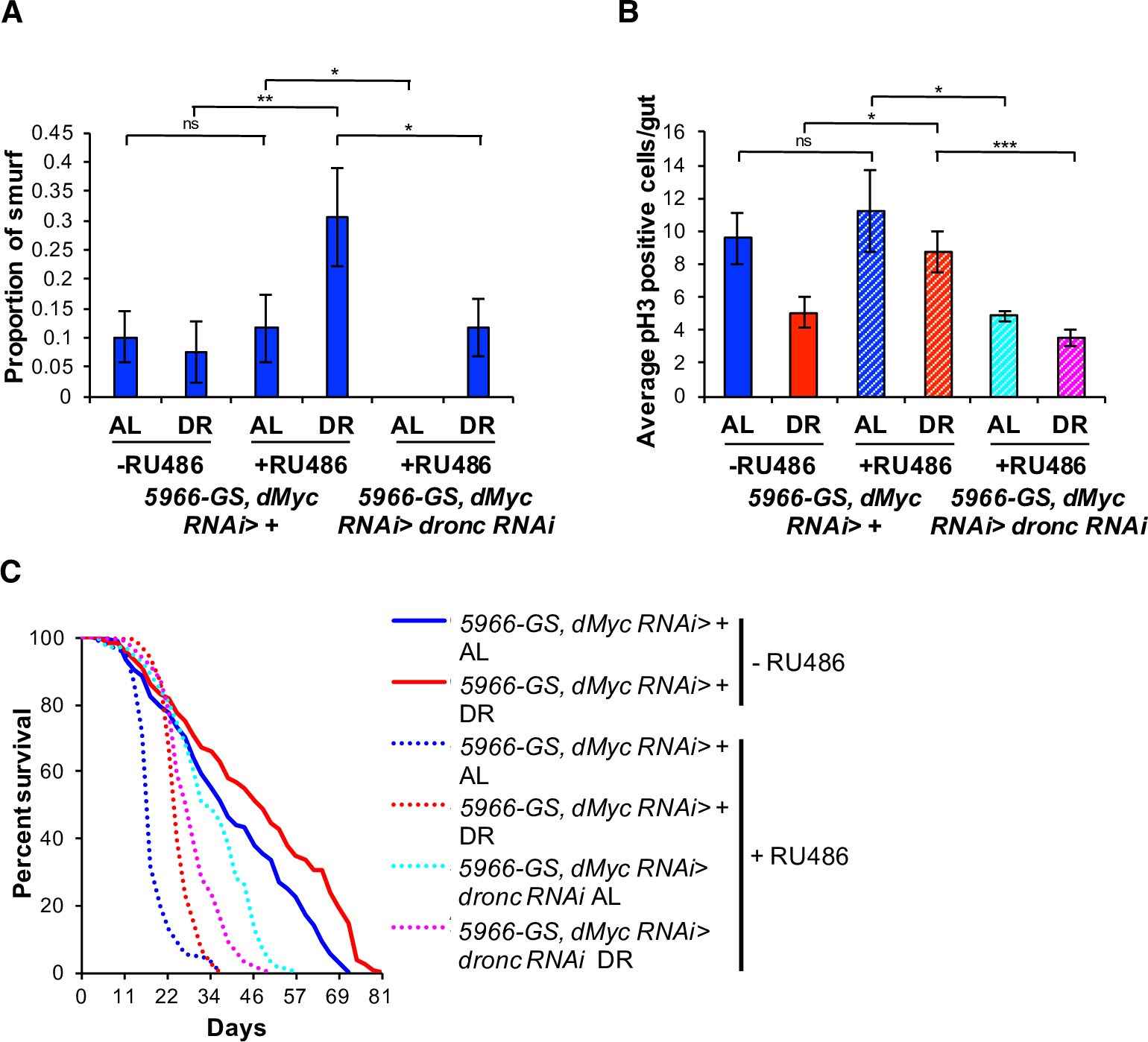

Supplement: S4 Fig — (A) Smurf gut permeability assay in 5966-GS, dMyc RNAi; + flies and 5966-GS, dMyc RNAi; dronc RNAi 20 days old flies. 5966-GS, dMyc RNAi; + flies–RU486 (AL: n = 61, DR: n = 51), +RU486 (AL: n = 40, DR: n = 70), 5966-GS, dMyc RNAi; dronc RNAi +RU486 (AL: n = 84, DR: n = 77). Error bars indicate SD of 4 different vials. (* p < 0.05 by t-test). (B) Mitotic ISCs quantification in 14 days old 5966-GS, dMyc RNAi; + and 5966-GS, dMyc RNAi; dronc RNAi flies. Error bars indicate SEM of 10 guts. (*** p < 0.001, * p < 0.05 by t-test). (C) Kaplan Meier survival analysis of 5966-GS, dMyc RNAi; + and 5966-GS, dMyc RNAi; dronc RNAi flies upon AL and DR. Statistical analysis of the survival curves and number of flies are provided in S2 Table. (TIF) [file pgen.1007777.s004.tif]

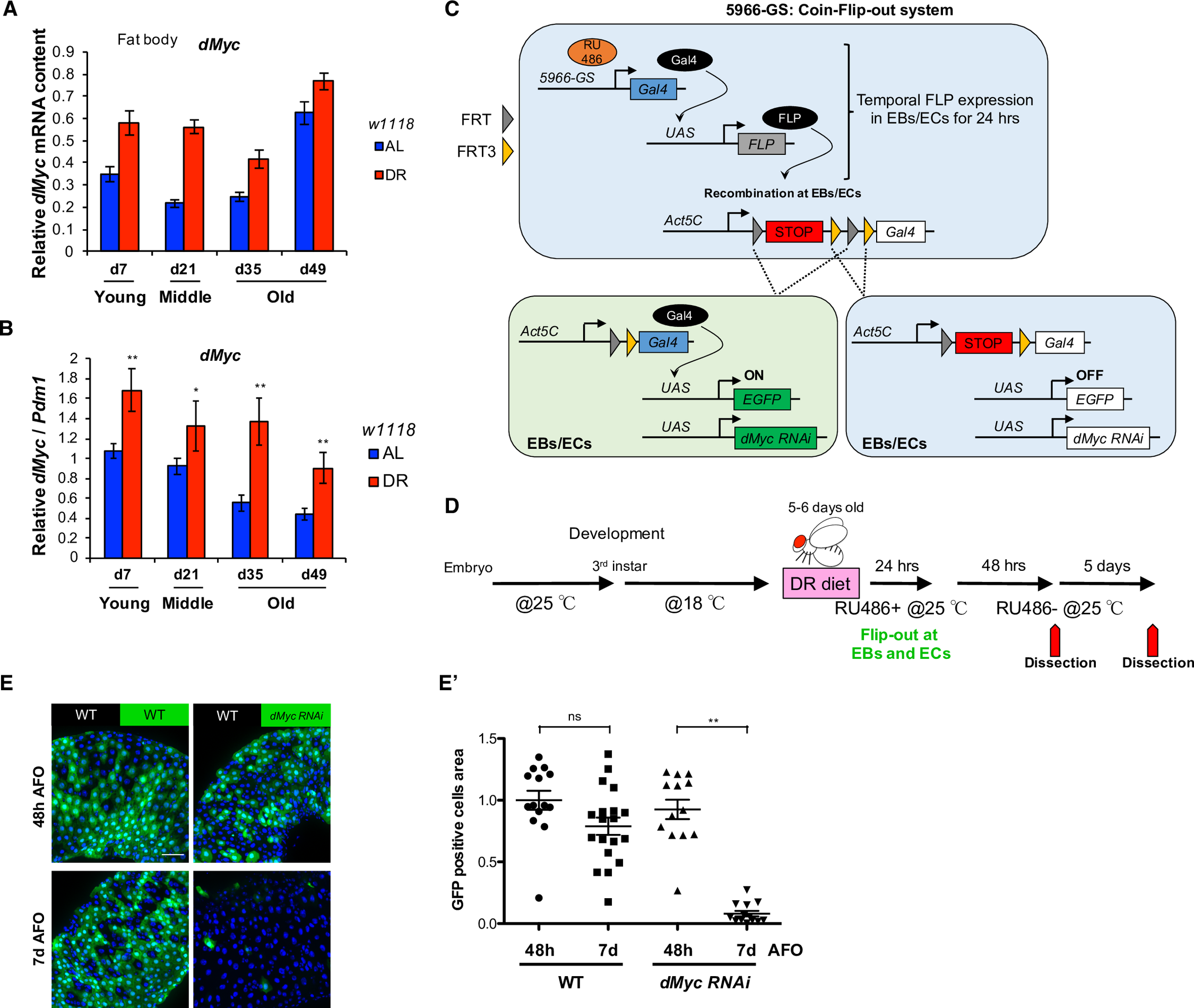

Supplement: S5 Fig — (A) dMyc mRNA expression in dissected fat bodies from w1118 upon AL and DR was measured with age. mRNA expression from flies at day 0 was set to 1. (B) dMyc mRNA expression in dissected guts from w1118 upon AL and DR was measured with age. dMyc mRNA expression was normalized by Pdm1 mRNA expression. mRNA expression from flies at day 0 was set to 1. (C) Schematic diagram for the 5966-GS: Coin-Flip-out system. CoinFLP-Gal4 system (Bosch et al., 2015) is utilized to induce dMyc RNAi mosaic cells in the post mitotic intestinal cells, EBs and ECs, as 5966-GS is allowed to express UAS-FLP in EBs and ECs during RU486 administration. Then, flies were maintained without RU486 and were dissected at 48 hours and 7 days after flip-out event (AFO). Flies were cultured at 18°C from 3rd instar larvae in order to reduce a leaky expression of Gal4. (D) Schematic diagram for the timeline of 5966-GS: Coin-Flip-out system. (E) GFP-positive flip-out EBs/ECs were observed at 48 hours (Top panels) and 7 days after flip-out (AFO) (Bottom panels) in the posterior midgut upon AL. (Left) WT flip-out EBs/ECs. (Right) dMyc RNAi flip-out EBs/ECs. Scale bar indicates 40 μm. (E’) Quantification of the size of GFP positive cells. (** p < 0.05 by t-test). (A and B) Error bars indicate SD of 3 independent biological replicates. (TIF) [file pgen.1007777.s005.tif]

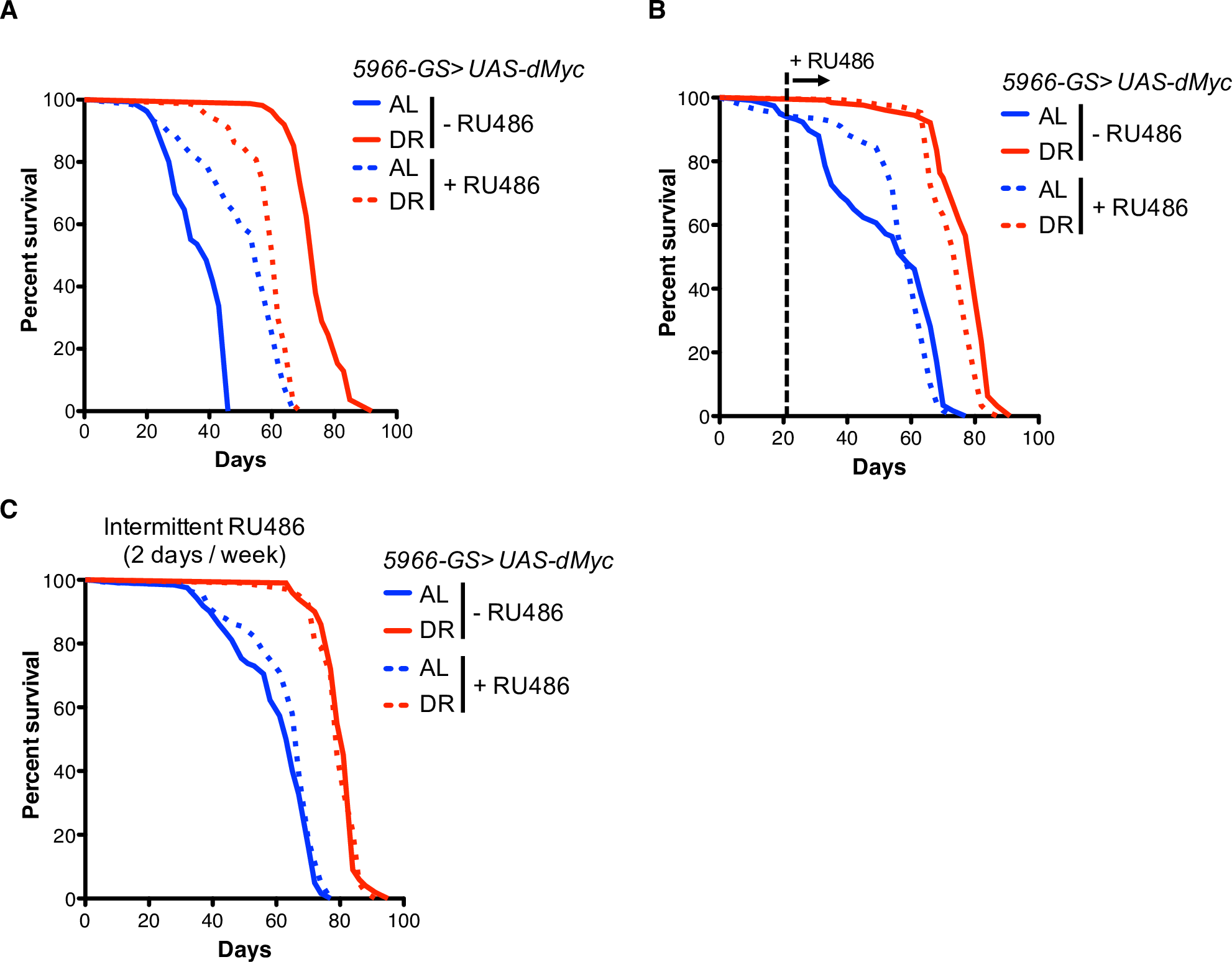

Supplement: S6 Fig — (A-C) Kaplan-Meier survival analysis of enteroblasts and enterocytes specific dMyc overexpression (5966-GS>UAS-dMyc) upon AL and DR. (A) RU486 was administrated from a day of sorting. (B) RU486 was administrated from day 21 of age. (C) RU486 was administrated every Monday and Tuesday during the adult stage. Statistical analysis of the survival curves and the number of flies are provided in S2 Table. (TIF) [file pgen.1007777.s006.tif]

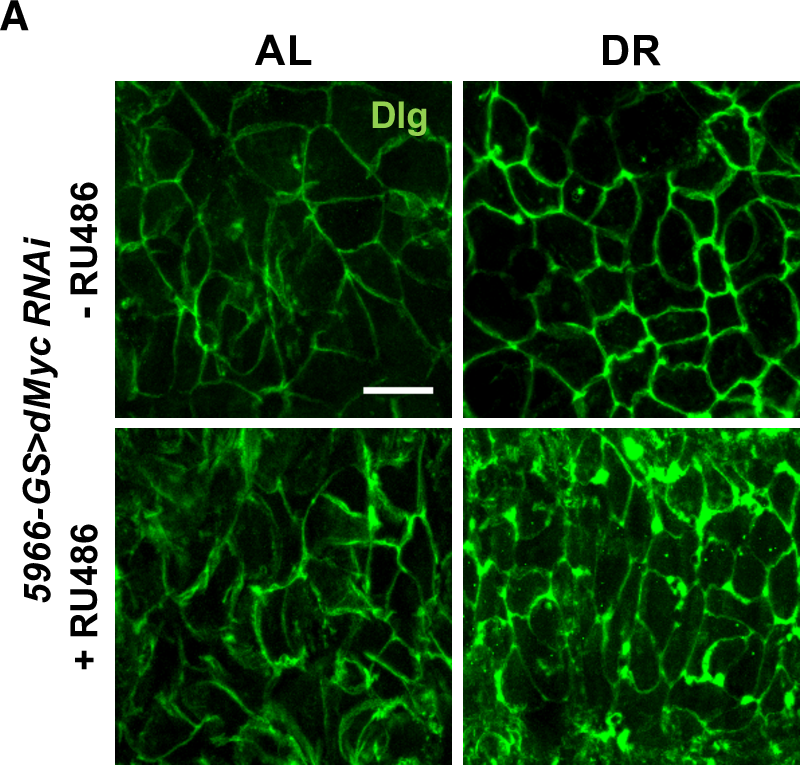

Supplement: S7 Fig — (A) Immunostaining of Discs large (Dlg) using dissected guts from 28 day old 5966-GS>dMyc RNAi flies. Representative image (n = 10). Scale bar indicates 20 μm. (TIF) [file pgen.1007777.s007.tif]
