# Supplementary material for: Dietary restriction improves intestinal cellular fitness to enhance gut barrier function and lifespan in D. melanogaster
Source: PLoS Genet. 2018 Nov 1;14(11):e1007777. doi: 10.1371/journal.pgen.1007777 (PMC6233930; doi:10.1371/journal.pgen.1007777)
Supplement: S1 Table — (DOCX) [file pgen.1007777.s008.docx]

**Table S1. Primer sets used for qRT-PCR.**

| Gene name | Forward primer (5’-3’) | Reverse primer (5’-3’) |
| --- | --- | --- |
| *rp 49* | CCACCAGTCGGATCGATATG | CACGTTGTGCACCAGGAACT |
| *dMyc* | AAATATCCTGCGAGAGGCGG | GAGCGCGATTCGTTCAACTC |
| *upd 3* | ACCTACAGAAGCGTTCCAG | GGTTCTGTAGATTCTGCAGG |
| *Diptericin* | ggcttatccgatgcccgacg | tctgtaggtgtaggtgcttccc |
| *puckered* | CGGGAACGGGGTAAATCCAA | GAGCAGTTACTACCCGCCAG |
| *hid* | CGATGTGTTCTTTCCGCACG | TGCTGCCGGAAGAAGTTGTA |
| *Pdm1* | cgggataaatcgaaggaagc | agtatttgatgtgtttgcgacttt |
