# Supplementary material for: Dietary restriction improves intestinal cellular fitness to enhance gut barrier function and lifespan in D. melanogaster
Source: PLoS Genet. 2018 Nov 1;14(11):e1007777. doi: 10.1371/journal.pgen.1007777 (PMC6233930; doi:10.1371/journal.pgen.1007777)
Supplement: S2 Table — (DOCX) [file pgen.1007777.s009.docx]

**Table S2. Statistical analysis of the survival curves.**

| Statistical analysis for Fig. 1A | | | | | | | |
| --- | --- | --- | --- | --- | --- | --- | --- |
| Group 1 / Group 2 | Chi square | P value | # of flies  Group 1 : Group 2 | | Median survival (MS) (days)  Group 1 : Group 2 | | % change in MS |
| Control DR / Control AL | 244.1 | < 0.0001 | 132 | 196 | 66 | 37 | +78 % |
|  |  |  |  |  |  |  |  |
| *5966-GS>dMyc RNAi* DR / *5966-GS>dMyc RNAi* AL | 158.8 | < 0.0001 | 134 | 193 | 45 | 30 | +50 % |
|  |  |  |  |  |  |  |  |
| *5966-GS>dMyc RNAi* DR / Control DR | 184.4 | < 0.0001 | 134 | 132 | 45 | 66 | -32 % |
|  |  |  |  |  |  |  |  |
| *5966-GS>dMyc RNAi* AL / Control AL | 74.34 | < 0.0001 | 193 | 196 | 30 | 37 | -19 % |

| Statistical analysis for Fig. 1B | | | | | | | |
| --- | --- | --- | --- | --- | --- | --- | --- |
| Group 1 / Group 2 | Chi square | P value | # of flies  Group 1 : Group 2 | | Median survival (MS) (days)  Group 1 : Group 2 | | % change in MS |
| Control DR / Control AL | 208.4 | < 0.0001 | 151 | 134 | 71 | 38 | +87 % |
|  |  |  |  |  |  |  |  |
| *5961-GS>dMyc RNAi* DR / *5961-GS>dMyc RNAi* AL | 186.0 | < 0.0001 | 152 | 130 | 62 | 29 | +114 % |
|  |  |  |  |  |  |  |  |
| *5961-GS>dMyc RNAi* DR / Control DR | 44.91 | < 0.0001 | 152 | 151 | 62 | 71 | -12 % |
|  |  |  |  |  |  |  |  |
| *5961-GS>dMyc RNAi* AL / Control AL | 29.95 | < 0.0001 | 130 | 134 | 29 | 38 | -24 % |

| Statistical analysis for Fig. 1C | | | | | | | |
| --- | --- | --- | --- | --- | --- | --- | --- |
| Group 1 / Group 2 | Chi square | P value | # of flies  Group 1 : Group 2 | | Median survival (MS) (days)  Group 1 : Group 2 | | % change in MS |
| Control DR / Control AL | 193.8 | < 0.0001 | 149 | 148 | 49 | 26 | +88 % |
|  |  |  |  |  |  |  |  |
| *S_1_106-GS>dMyc RNAi* DR / *S_1_106-GS>dMyc RNAi* AL | 196.3 | < 0.0001 | 145 | 148 | 49 | 21 | +133 % |
|  |  |  |  |  |  |  |  |
| *S_1_106-GS>dMyc RNAi* DR / Control DR | 19.54 | < 0.0001 | 145 | 149 | 49 | 49 | 0 % |
|  |  |  |  |  |  |  |  |
| *S_1_106-GS>dMyc RNAi* AL / Control AL | 16.91 | < 0.0001 | 148 | 148 | 21 | 26 | -19 % |

| Statistical analysis for Fig. 3F | | | | | | | |
| --- | --- | --- | --- | --- | --- | --- | --- |
| Group 1 / Group 2 | Chi square | P value | # of flies  Group 1 : Group 2 | | Median survival (MS) (days)  Group 1 : Group 2 | | % change in MS |
| Control DR / Control AL | 41.37 | < 0.0001 | 120 | 110 | 64 | 56 | +14 % |
|  |  |  |  |  |  |  |  |
| *5966-GS, dMyc RNAi>+* DR / Control DR | 273.0 | < 0.0001 | 164 | 120 | 27 | 64 | -58 % |
|  |  |  |  |  |  |  |  |
| *5966-GS, dMyc RNAi>+* AL / Control AL | 201.5 | < 0.0001 | 167 | 110 | 20 | 56 | -64 % |
|  |  |  |  |  |  |  |  |
| *5966-GS, dMyc RNAi>UAS-p35* DR / *5966-GS, dMyc RNAi>+* DR | 81.81 | < 0.0001 | 164 | 164 | 36 | 27 | +33 % |
|  |  |  |  |  |  |  |  |
| *5966-GS, dMyc RNAi>UAS-p35* AL / *5966-GS, dMyc RNAi>+* AL | 89.37 | < 0.0001 | 161 | 167 | 24 | 20 | +20% |

| Statistical analysis for Fig. 4G | | | | | | | |
| --- | --- | --- | --- | --- | --- | --- | --- |
| Group 1 / Group 2 | Chi square | P value | # of flies  Group 1 : Group 2 | | Median survival (MS) (days)  Group 1 : Group 2 | | % change in MS |
| Control AL (+AB) / Control AL (-AB) | 108.6 | < 0.0001 | 76 | 146 | 62 | 24 | +158 % |
|  |  |  |  |  |  |  |  |
| *5966-GS>dMyc RNAi* DR (-AB) / *5966-GS>dMyc RNAi* AL (-AB) | 36.64 | < 0.0001 | 119 | 112 | 27 | 22 | +23 % |
|  |  |  |  |  |  |  |  |
| *5966-GS>dMyc RNAi* DR (+AB) / *5966-GS>dMyc RNAi* AL (+AB) | 202.3 | < 0.0001 | 114 | 111 | 41 | 22 | +86 % |
|  |  |  |  |  |  |  |  |
| *5966-GS>dMyc RNAi* DR (+AB) / *5966-GS>dMyc RNAi* DR (-AB) | 109.7 | < 0.0001 | 114 | 119 | 41 | 27 | +52 % |
|  |  |  |  |  |  |  |  |
| *5966-GS>dMyc RNAi* AL (+AB) / *5966-GS>dMyc RNAi* AL (-AB) | 0.50 | 0.48 | 111 | 112 | 22 | 22 | 0 % |

| Statistical analysis for Fig. 6A | | | | | | | |
| --- | --- | --- | --- | --- | --- | --- | --- |
| Group 1 / Group 2 | Chi square | P value | # of flies  Group 1 : Group 2 | | Median survival (MS) (days)  Group 1 : Group 2 | | % change in MS |
| Control DR / Control AL | 198.0 | < 0.0001 | 193 | 198 | 76 | 55 | +38 % |
|  |  |  |  |  |  |  |  |
| *5966-GS>UAS-dMyc* DR / *5966-GS>UAS-dMyc* AL | 301.2 | < 0.0001 | 165 | 152 | 83 | 57 | +46 % |
|  |  |  |  |  |  |  |  |
| *5966-GS>UAS-dMyc* DR / Control DR | 5.84 | 0.0157 | 165 | 193 | 83 | 76 | +9 % |
|  |  |  |  |  |  |  |  |
| *5966-GS>UAS-dMyc* AL / Control AL | 4.789 | 0.0286 | 152 | 198 | 57 | 55 | +4 % |

| Statistical analysis for Fig. 6B | | | | | | | |
| --- | --- | --- | --- | --- | --- | --- | --- |
| Group 1 / Group 2 | Chi square | P value | # of flies  Group 1 : Group 2 | | Median survival (MS) (days)  Group 1 : Group 2 | | % change in MS |
| Control DR / Control AL | 103.4 | < 0.0001 | 104 | 94 | 81 | 65 | +25 % |
|  |  |  |  |  |  |  |  |
| *5966-GS>UAS-dMyc* DR / *5966-GS>UAS-dMyc* AL | 199.1 | < 0.0001 | 109 | 123 | 84 | 70 | +20 % |
|  |  |  |  |  |  |  |  |
| *5966-GS>UAS-dMyc* DR / Control DR | 9.433 | 0.0021 | 109 | 104 | 84 | 81 | +1 % |
|  |  |  |  |  |  |  |  |
| *5966-GS>UAS-dMyc* AL / Control AL | 11.02 | 0.0009 | 123 | 94 | 70 | 65 | +8 % |

| Statistical analysis for Fig. S1A | | | | | | | |
| --- | --- | --- | --- | --- | --- | --- | --- |
| Group 1 / Group 2 | Chi square | P value | # of flies  Group 1 : Group 2 | | Median survival (MS) (days)  Group 1 : Group 2 | | % change in MS |
| Control DR / Control AL | 215.5 | < 0.0001 | 179 | 168 | 73 | 35 | + 109 % |
|  |  |  |  |  |  |  |  |
| *5966-GS>dMyc RNAi^TRiP-1^* DR / *5966-GS>dMyc RNAi^TRiP-1^* AL | 230.4 | < 0.0001 | 169 | 181 | 59 | 24 | +146 % |
|  |  |  |  |  |  |  |  |
| *5966-GS>dMyc RNAi^TRiP-1^* DR / Control DR | 132.2 | < 0.0001 | 169 | 179 | 59 | 73 | -19 % |
|  |  |  |  |  |  |  |  |
| *5966-GS>dMyc RNAi^TRiP-1^* AL / Control AL | 22.74 | < 0.0001 | 181 | 168 | 24 | 35 | -31 % |

| Statistical analysis for Fig. S1B | | | | | | | |
| --- | --- | --- | --- | --- | --- | --- | --- |
| Group 1 / Group 2 | Chi square | P value | # of flies  Group 1 : Group 2 | | Median survival (MS) (days)  Group 1 : Group 2 | | % change in MS |
| Control DR / Control AL | 98.58 | < 0.0001 | 173 | 183 | 56 | 33 | +70 % |
|  |  |  |  |  |  |  |  |
| *5966-GS>dMyc RNAi^TRiP-2^* DR / *5966-GS>dMyc RNAi^TRiP-2^* AL | 45.76 | < 0.0001 | 185 | 180 | 45 | 40 | + 13% |
|  |  |  |  |  |  |  |  |
| *5966-GS>dMyc RNAi^TRiP-2^* DR / Control DR | 86.98 | < 0.0001 | 185 | 173 | 45 | 56 | -20 % |
|  |  |  |  |  |  |  |  |
| *5966-GS>dMyc RNAi^TRiP-2^* AL / Control AL | 6.587 | 0.0103 | 180 | 183 | 40 | 33 | +21 % |

| Statistical analysis for Fig. S1C | | | | | | | |
| --- | --- | --- | --- | --- | --- | --- | --- |
| Group 1 / Group 2 | Chi square | P value | # of flies  Group 1 : Group 2 | | Median survival (MS) (days)  Group 1 : Group 2 | | % change in MS |
| *Np-1, tub-Gal80ts>*+ DR / *Np-1, tub-Gal80ts>*+ AL | 40.47 | < 0.0001 | 119 | 118 | 42 | 39 | +8 % |
|  |  |  |  |  |  |  |  |
| *dMyc RNAi>+* DR / *dMyc RNAi>+* AL | 175.6 | < 0.0001 | 185 | 165 | 37 | 32 | +16 % |
|  |  |  |  |  |  |  |  |
| *Np-1, tub-Gal80ts>dMyc RNAi* DR / *Np-1, tub-Gal80ts >dMyc RNAi* AL | 18.88 | < 0.0001 | 138 | 149 | 32 | 29 | +10 % |
|  |  |  |  |  |  |  |  |
| *Np-1, tub-Gal80ts>dMyc RNAi* DR / *Np-1, tub-Gal80ts>*+ DR | 122.9 | < 0.0001 | 138 | 119 | 32 | 42 | -24 % |
|  |  |  |  |  |  |  |  |
| *Np-1, tub-Gal80ts>dMyc RNAi* DR / *dMyc RNAi>+* DR | 87.39 | < 0.0001 | 138 | 185 | 32 | 37 | -14 % |
|  |  |  |  |  |  |  |  |
| *Np-1, tub-Gal80ts>dMyc RNAi* AL / *Np-1, tub-Gal80ts>*+ AL | 102.3 | < 0.0001 | 149 | 118 | 29 | 39 | -26 % |
|  |  |  |  |  |  |  |  |
| *Np-1, tub-Gal80ts>dMyc RNAi* AL / *dMyc RNAi>+* AL | 10.94 | 0.0009 | 149 | 165 | 29 | 32 | -9 % |

| Statistical analysis for Fig. S1D | | | | | | | |
| --- | --- | --- | --- | --- | --- | --- | --- |
| Group 1 / Group 2 | Chi square | P value | # of flies  Group 1 : Group 2 | | Median survival (MS) (days)  Group 1 : Group 2 | | % change in MS |
| Control DR / Control AL | 313.9 | < 0.0001 | 148 | 175 | 78 | 46 | +70 % |
|  |  |  |  |  |  |  |  |
| *Act5C-GS>dMyc RNAi* DR / *Act5C-GS>dMyc RNAi* AL | 56.77 | < 0.0001 | 183 | 157 | 46 | 29 | +59 % |
|  |  |  |  |  |  |  |  |
| *Act5C-GS>dMyc RNAi* DR / Control DR | 356.4 | < 0.0001 | 183 | 148 | 46 | 78 | -41 % |
|  |  |  |  |  |  |  |  |
| *Act5C-GS>dMyc RNAi* AL / Control AL | 71.57 | < 0.0001 | 157 | 175 | 29 | 46 | -37 % |

| Statistical analysis for Fig. S3D | | | | | | | |
| --- | --- | --- | --- | --- | --- | --- | --- |
| Group 1 / Group 2 | Chi square | P value | # of flies  Group 1 : Group 2 | | Median survival (MS) (days)  Group 1 : Group 2 | | % change in MS |
| Control DR / Control AL | 10.07 | 0.0015 | 50 | 43 | 40 | 32 | +25 % |
|  |  |  |  |  |  |  |  |
| *5966-GS, dMyc RNAi>+* DR / Control DR | 42.65 | < 0.0001 | 163 | 50 | 29 | 40 | -28 % |
|  |  |  |  |  |  |  |  |
| *5966-GS, dMyc RNAi>+* AL / Control AL | 45.68 | < 0.0001 | 165 | 43 | 19 | 32 | -41 % |
|  |  |  |  |  |  |  |  |
| *5966-GS, dMyc RNAi>UAS-Bsk^DN^* DR / *5966-GS, dMyc RNAi>+* DR | 7.193 | 0.0073 | 165 | 163 | 26 | 29 | -10% |
|  |  |  |  |  |  |  |  |
| *5966-GS, dMyc RNAi>UAS-Bsk^DN^* AL / *5966-GS, dMyc RNAi>+* AL | 0.3868 | 0.5340 | 156 | 165 | 22 | 19 | +16 % |

| Statistical analysis for Fig. S3E | | | | | | | |
| --- | --- | --- | --- | --- | --- | --- | --- |
| Group 1 / Group 2 | Chi square | P value | # of flies  Group 1 : Group 2 | | Median survival (MS) (days)  Group 1 : Group 2 | | % change in MS |
| Control DR / Control AL | 65.80 | < 0.0001 | 184 | 177 | 63 | 49 | +29 % |
|  |  |  |  |  |  |  |  |
| *5966-GS>UAS-Bsk^DN^* DR / *5966-GS>UAS-Bsk^DN^* AL | 332.8 | < 0.0001 | 186 | 192 | 49 | 31 | +58 % |
|  |  |  |  |  |  |  |  |
| *5966-GS>UAS-Bsk^DN^* DR / Control DR | 142.9 | < 0.0001 | 186 | 184 | 49 | 63 | -22% |
|  |  |  |  |  |  |  |  |
| *5966-GS>UAS-Bsk^DN^* AL / Control AL | 276.0 | < 0.0001 | 192 | 177 | 31 | 49 | -37% |

| Statistical analysis for Fig. S4C | | | | | | | |
| --- | --- | --- | --- | --- | --- | --- | --- |
| Group 1 / Group 2 | Chi square | P value | # of flies  Group 1 : Group 2 | | Median survival (MS) (days)  Group 1 : Group 2 | | % change in MS |
| Control DR / Control AL | 21.58 | < 0.0001 | 151 | 166 | 48 | 39 | +23 % |
|  |  |  |  |  |  |  |  |
| *5966-GS, dMyc RNAi>+* DR / Control DR | 136.5 | < 0.0001 | 159 | 151 | 25 | 48 | -48 % |
|  |  |  |  |  |  |  |  |
| *5966-GS, dMyc RNAi>+* AL / Control AL | 172.1 | < 0.0001 | 163 | 166 | 18 | 39 | -54 % |
|  |  |  |  |  |  |  |  |
| *5966-GS, dMyc RNAi>dronc RNAi* DR / *5966-GS, dMyc RNAi>+* DR | 36.35 | < 0.0001 | 162 | 159 | 29 | 25 | +16 % |
|  |  |  |  |  |  |  |  |
| *5966-GS, dMyc RNAi> dronc RNAi* AL / *5966-GS, dMyc RNAi>+* AL | 183.7 | < 0.0001 | 156 | 163 | 34 | 18 | +89 % |

| Statistical analysis for Fig. S6A | | | | | | | |
| --- | --- | --- | --- | --- | --- | --- | --- |
| Group 1 / Group 2 | Chi square | P value | # of flies  Group 1 : Group 2 | | Median survival (MS) (days)  Group 1 : Group 2 | | % change in MS |
| Control DR / Control AL | 333.9 | < 0.0001 | 163 | 136 | 74 | 39 | + 90 % |
|  |  |  |  |  |  |  |  |
| *5966-GS>UAS-dMyc* DR / *5966-GS>UAS-dMyc* AL | 31.93 | < 0.0001 | 146 | 133 | 62 | 55 | +13 % |
|  |  |  |  |  |  |  |  |
| *5966-GS>UAS-dMyc* DR / Control DR | 266.4 | < 0.0001 | 146 | 163 | 62 | 74 | - 16 % |
|  |  |  |  |  |  |  |  |
| *5966-GS>UAS-dMyc* AL / Control AL | 108.3 | < 0.0001 | 133 | 136 | 55 | 39 | +41 % |

| Statistical analysis for Fig. S6B | | | | | | | |
| --- | --- | --- | --- | --- | --- | --- | --- |
| Group 1 / Group 2 | Chi square | P value | # of flies  Group 1 : Group 2 | | Median survival (MS) (days)  Group 1 : Group 2 | | % change in MS |
| Control DR / Control AL | 179.2 | < 0.0001 | 127 | 117 | 80 | 59 | +36 % |
|  |  |  |  |  |  |  |  |
| *5966-GS>UAS-dMyc* DR / *5966-GS>UAS-dMyc* AL | 203.7 | < 0.0001 | 149 | 121 | 75 | 59 | +27 % |
|  |  |  |  |  |  |  |  |
| *5966-GS>UAS-dMyc* DR / Control DR | 29.93 | < 0.0001 | 149 | 127 | 75 | 80 | -6 % |
|  |  |  |  |  |  |  |  |
| *5966-GS>UAS-dMyc* AL / Control AL | 3.067 | 0.0799 | 121 | 117 | 59 | 59 | 0 % |

| Statistical analysis for Fig. S6C | | | | | | | |
| --- | --- | --- | --- | --- | --- | --- | --- |
| Group 1 / Group 2 | Chi square | P value | # of flies  Group 1 : Group 2 | | Median survival (MS) (days)  Group 1 : Group 2 | | % change in MS |
| Control DR / Control AL | 196.2 | < 0.0001 | 100 | 122 | 81 | 64 | +27 % |
|  |  |  |  |  |  |  |  |
| *5966-GS>UAS-dMyc* DR / *5966-GS>UAS-dMyc* AL | 173.6 | < 0.0001 | 104 | 124 | 79 | 67 | +18 % |
|  |  |  |  |  |  |  |  |
| *5966-GS>UAS-dMyc* DR / Control DR | 0.4061 | 0.5240 | 104 | 100 | 79 | 81 | -2 % |
|  |  |  |  |  |  |  |  |
| *5966-GS>UAS-dMyc* AL / Control AL | 4.347 | 0.0371 | 124 | 122 | 67 | 64 | +5 % |
