# Supplementary material for: Dietary restriction improves intestinal cellular fitness to enhance gut barrier function and lifespan in D. melanogaster
Source: PLoS Genet. 2018 Nov 1;14(11):e1007777. doi: 10.1371/journal.pgen.1007777 (PMC6233930; doi:10.1371/journal.pgen.1007777)
Supplement: S3 Table — (DOCX) [file pgen.1007777.s010.docx]

**Table S3. Summary of the independent repeats of the lifespan analysis.**

| The independent repeat lifespan analysis of Fig. 1A | | | | |
| --- | --- | --- | --- | --- |
|  | Median survival (days) | | | |
| Repeat # | Control DR (n) | Control AL (n) | *5966-GS>dMyc RNAi* DR (n) | *5966-GS>dMyc RNAi* AL (n) |
| 1 | 48 (128) | 25 (146) | 36 (117) | 20 (168) |
|  | +92 % (P < 0.0001) | | +80 % (P < 0.0001) | |
| 2 | 55 (150) | 26 (146) | 39 (134) | 23 (148) |
|  | +112 % (P < 0.0001) | | +70 % (P < 0.0001) | |

| The independent repeat lifespan analysis of Fig. 1B | | | | |
| --- | --- | --- | --- | --- |
|  | Median survival (days) | | | |
| Repeat # | Control DR (n) | Control AL (n) | *5961-GS>dMyc RNAi* DR (n) | *5961-GS>dMyc RNAi* AL (n) |
| 1 | 58 (135) | 28 (127) | 62 (115) | 28 (131) |
|  | +107 % (P < 0.0001) | | +121 % (P < 0.0001) | |

| The independent repeat lifespan analysis of Fig. 1C | | | | |
| --- | --- | --- | --- | --- |
|  | Median survival (days) | | | |
| Repeat # | Control DR (n) | Control AL (n) | *S_1_106-GS>dMyc RNAi* DR (n) | *S_1_106-GS>dMyc RNAi* AL (n) |
| 1 | 50 (154) | 22 (108) | 53 (134) | 23 (85) |
|  | +127 % (P < 0.0001) | | +130 % (P < 0.0001) | |

| The independent repeat lifespan analysis of Fig. 3F | | | | | | |
| --- | --- | --- | --- | --- | --- | --- |
|  | Median survival (days) | | | | | |
| Repeat # | Control AL (n) | Control AL (n) | *5966-GS,dMyc RNAi>UAS-p35* DR (n) | *5966-GS,dMyc RNAi>+* DR (n) | *5966-GS,dMyc RNAi>UAS-p35* AL (n) | *5966-GS,dMyc RNAi>+* AL (n) |
| 1 | 64 (95) | 77 (114) | 34 (107) | 24 (101) | 48 (115) | 21 (118) |
|  | +20 % (P < 0.0001) | | +42 % (P < 0.0001) | | +129 % (P < 0.0001) | |
| 2 | 58 (160) | 60 (138) | 42 (153) | 24 (162) | 49 (168) | 21 (155) |
|  | +4% (P=0.0081) | | +75 % (P < 0.0001) | | +133 % (P < 0.0001) | |

| The independent repeat lifespan analysis of Fig. 4G | | | | | | |
| --- | --- | --- | --- | --- | --- | --- |
|  | Median survival (days) | | | | | |
| Repeat # | Control AL (-AB) (n) | Control AL (+AB) (n) | *5966-GS>dMyc RNAi* DR (-AB) (n) | *5966-GS>dMyc RNAi* AL (-AB) (n) | *5966-GS>dMyc RNAi* DR (+AB) (n) | *5966-GS>dMyc RNAi* AL (+AB) (n) |
| 1 | 64 (95) | 77 (114) | 34 (107) | 24 (101) | 48 (115) | 21 (118) |
|  | +20 % (P < 0.0001) | | +42 % (P < 0.0001) | | +129 % (P < 0.0001) | |
| 2 | 58 (160) | 60 (138) | 42 (153) | 24 (162) | 49 (168) | 21 (155) |
|  | +4% (P=0.0081) | | +75 % (P < 0.0001) | | +133 % (P < 0.0001) | |
