# Supplementary material for: Dietary restriction improves intestinal cellular fitness to enhance gut barrier function and lifespan in D. melanogaster
Source: PLoS Genet. 2018 Nov 1;14(11):e1007777. doi: 10.1371/journal.pgen.1007777 (PMC6233930; doi:10.1371/journal.pgen.1007777)
Supplement: S4 Table — (DOCX) [file pgen.1007777.s011.docx]

**Table S4. The statistical analysis for EB/EC or ISC/EB-specific *dMyc* knockdown lifespan.**

| Cox proportional-hazards ratio^a^ statistical analysis for EB/EC or ISC/EB-specific *dMyc* knockdown lifespan | | | | | |
| --- | --- | --- | --- | --- | --- |
| coxph(formula = Surv(time, status) ~ diet^b^ * gene effect^c^) | | | | | |
| Genotype | Predictor | Hazard Ratio (HR) | Wald statistic *p* value for predictor coefficient | Number of flies (n) | Model Likelihood ratio test *p* value |
| *5966-GS>dMyc RNAi*  (Fig. 1A) | diet | 0.07734 | < 2e-16*** | 655 | <2e-16 |
|  | gene effect | 2.86658 | < 2e-16*** |  |  |
|  | diet:gene effect interaction | 2.70895 | 0.000000406*** |  |  |
| *5966-GS>dMyc RNAi^TRiP-1^*  (Fig. S1A) | diet | 0.1172 | < 2e-16*** | 695 | <2e-16 |
|  | gene effect | 2.2464 | 0.00000000000289*** |  |  |
|  | diet:gene effect interaction | 1.8435 | 0.000381*** |  |  |
| *5966-GS>dMyc RNAi^TRiP-2^*  (Fig. S1B) | diet | 0.2791 | < 2e-16*** | 721 | <2e-16 |
|  | gene effect | 1.4777 | 0.000415*** |  |  |
|  | diet:gene effect interaction | 2.072 | 0.00000343*** |  |  |
| *5961-GS>dMyc RNAi*  (Fig. 1B) | diet | 0.06946 | < 2e-16*** | 567 | <2e-16 |
|  | gene effect | 1.77319 | 0.00000829*** |  |  |
|  | diet:gene effect interaction | 1.2965 | 0.145 |  |  |
| Signif. codes: 0 ‘***’, 0.001 ‘**’, 0.01 ‘*’, 0.05 ‘.’, 0.1 ‘ ’, 1 | | | | | |
| ^a^Statistical analyses (cox proportional-hazards ratio model) were performed using the R 3.3.3 software | | | | | |
| ^b^diet (AL = 0, DR = 1) | | | | | |
| ^c^gene effect (without = 0, with = 1) | | | | | |
